# Editorial

**Published:** 1914-12

**Authors:** 


### EDITORIAL

The Business Office of the *Journal-Record* is 23 West Alabama Street.

The Editorial Office is 1014-15 Atlanta National Bank Building.

Address all Business Communications to *Journal-Record of Medicine*, 23 West Alabama Street.

Make remittances payable to *The Journal-Record of Medicine.*

On matters pertaining to the Editorial and Original Communications, address Edgar G. Ballenger, M. D., Atlanta, Ga.

Reprints of Original Articles will be furnished at cost price. Requests for the same should always be made in THE MANUSCRIPT.

We will present, postpaid, on request to each contributor of an original article, twenty (20) marked copies of *The Journal-Record of Medicine* containing such article.

### INTRADURAL MEDICATION.

The interest now taken in the treatment of syphilis and especially syphilis of the nervous system has brought out new methods of applying remedies to the spinal cord, namely, by intradural injections. Many more or less favorable reports have indicated that this method of treatment affords more satisfactory results than when the remedies are given intravenously. At first thought such an avenue appeals to one as being rational, and the early reports gave apparently added weight to the advantage of this procedure. A careful digest of the reports and an unbiased consideration of the subject, however, makes one hesitate as to the final acceptance of the method. The Swift and Ellis plan is the injection of salvarsan intravenously and within an hour to withdraw blood, separate the serum and inject about 40 c. c. into the dura after withdrawing a similar amount of spinal fluid. Very small amounts of neosalvarsan may be injected directly into spinal cavity. Both of these methods are more dangerous than the intravenous injections of salvarsan and neosalvarsan. Reported injections of these remedies appear to give about the same results as do the intradural injections. Nearly all the good reports of the intradural method have been duplicated by the repeated intravenous injections. When taken early nervous affections respond as a rule to either method of treatment. When seen very late with extensive damage already done to the nervous system neither method can affect much, if any, good. Between these extremes are patients who may improve or in whom the process may be checked. To assert that the salvarsan does not reach the nervous system on account the poor circulation is not in accord with reason or facts. The effect of morphine, alcohol, chloroform, ether, bromides, strychnine, and many other drugs strongly contradict such an idea. Any portion of the nervous system not reached by blood or lymph must undergo necrosis like any other part of the body, except the cornea. That the spinal fluid itself does not receive much of the salvarsan has little bearing on the subject for the trouble is not with the fluid but with the nerves suspended in it and we know they do not absorb it as a sponge. It is the body of the nerve, not its surface, bathed in the spinal fluid, that is affected. That the cell content becomes normal more rapidly after intradural injections than after intravenous alone will be granted. The lymphocytosis is not the disease, however, but merely the expression of a small part of it, that is, the meningeal surface. Even this usually responds to repeated intravenous injections if the process is not too far advanced to hope for improvement from either procedure. Since there is a possibility of securing good results without the intradural injections and the danger to the patient is greatly increased by them, we would urge that they not be employed except when treatment with repeated intravenous medication supplemented with intensive courses of treatment with mercury have failed.

---

### GEORGIA SURGEON'S CLUB.

The Georgia Surgeons' Club will hold a meeting in Atlanta on the 25th and 26th of February. Clinics are being arranged for these two days so that those attending the meeting will have a large variety from which to select the work they wish to see.

The surgeons who are to give these clinics are very much interested in the coming meeting and are making the necessary plans to demonstrate their best work, which will reflect the modern and improved methods of dealing with surgical affections. On the evening of the 25th a subscription banquet will be given at one of the leading hotels; the program will include a symposium on "Surgical Indigestion."

Altogether the meeting will be one of the most interesting and instructive that has been held in Atlanta. A large attendance is expected. A cordial invitation is extended to the doctors of this territory and all who come will receive a hearty welcome.

